# Microalgae: Green Engines for Achieving Carbon Sequestration, Circular Economy, and Environmental Sustainability—A Review Based on Last Ten Years of Research

**DOI:** 10.3390/bioengineering12090909

**Published:** 2025-08-25

**Authors:** Md. Muzammal Hoque, Valeria Iannelli, Francesca Padula, Rosa Paola Radice, Biplob Kumar Saha, Giuseppe Martelli, Antonio Scopa, Marios Drosos

**Affiliations:** 1 Department of Agricultural, Forestry, Food and Environmental Sciences (DAFE), University of Basilicata, Viale dell’Ateneo Lucano 10, 85100 Potenza, Italy; md.muzammal.hoque@unibas.it (M.M.H.); valeria.iannelli@unibas.it (V.I.); antonio.scopa@unibas.it (A.S.); 2 Bangladesh Institute of Nuclear Agriculture (BINA), Mymensingh 2202, Bangladesh; 3 AlgaeBioMed srl, via Luigi Kossuth 7, 00149 Roma, Italy; 4 Department of Basic and Applied Science (DiSBA), University of Basilicata, Viale dell’Ateneo Lucano 10, 85100 Potenza, Italy; francesca.padula@unibas.it (F.P.); rosapaolaradice@gmail.com (R.P.R.); giuseppe.martelli@unibas.it (G.M.); 5 Bioinnova srls, via Ponte Nove Luci 22, 85100 Potenza, Italy; 6 Department of Agricultural Chemistry, Bangladesh Agricultural University, Mymensingh 2202, Bangladesh; bkasaha@bau.edu.bd

**Keywords:** microalgae, soil health, carbon sequestration, climate change mitigation, sustainable agriculture, circular economy

## Abstract

Feeding a growing global population requires sustainable, innovative, and cost-effective solutions, especially in light of the environmental damage and nutrient imbalances caused by excessive chemical fertilizer use. Microalgae have gained prominence due to their phylogenetic diversity, physiological adaptability, eco-compatible characteristics, and potential to support regenerative agriculture and mitigate climate change. Functioning as biofertilizers, biostimulants, and bioremediators, microalgae accelerate nutrient cycling, improve soil aggregation through extracellular polymeric substances (EPSs), and stimulate rhizospheric microbial diversity. Empirical studies demonstrate their ability to increase crop yields by 5–25%, reduce chemical nitrogen inputs by up to 50%, and boost both organic carbon content and enzymatic activity in soils. Their application in saline and degraded lands further promotes resilience and ecological regeneration. Microalgal cultivation platforms offer scalable in situ carbon sequestration, converting atmospheric carbon dioxide (CO_2_) into biomass with potential downstream vaporization into biofuels, bioplastics, and biochar, aligning with circular economy principles. While the commercial viability of microalgae is challenged by high production costs, technical complexities, and regulatory gaps, recent breakthroughs in cultivation systems, biorefinery integration, and strain optimization highlight promising pathways forward. This review highlights the strategic importance of microalgae in enhancing climate resilience, promoting agricultural sustainability, restoring soil health, and driving global bioeconomic transformation.

## 1. Introduction

Soil is a complex ecosystem that hosts a vast array of microbes, which play a crucial role in decomposing organic matter and driving biogeochemical nutrient cycles [1]. Modern agriculture faces significant challenges due to the uncontrolled use of synthetic agrochemicals aimed at boosting crop yields [2]. Environmentally friendly bio-inoculants, such as symbiotic nitrogen (N) fixers, rhizobacteria, mycorrhizae, and microalgae, offer a sustainable alternative, delivering multiple benefits to plants and reducing the reliance on synthetic inputs [3,4,5]. Agricultural practices and conventional farming systems are under increasing pressure due to global issues such as climate change, rapid population growth, and environmental degradation. This highlights the pressing need for sustainable solutions that improve resource efficiency and mitigate ecological impacts [6]. Within this context, microalgae have gained attention for their potential contributions to carbon sequestration, climate change mitigation, soil health improvement, and sustainable farming systems [7,8,9].

Microalgal biomass enhances soil health by improving structure, increasing water retention capacity, stimulating microbial activity, and supplying essential nutrients [9]. These microorganisms possess a remarkable ability to absorb CO_2_ and nutrients from wastewater, thereby boosting soil fertility [10]. Their integration into agricultural systems presents a viable alternative to chemical fertilizers, offering economic benefits and supporting the overall nitrogen cycle [11,12]. Bioactive compounds produced by microalgae [13] act as biostimulants, promoting microbial activity, nutrient cycling, and plant development by releasing growth hormones, polysaccharides, and antimicrobial substances [9]. Thanks to their adaptability, microalgae are also ideal candidates for the sustainable production of biofuels and other high-value products such as bioplastics, pharmaceuticals, and nutraceuticals [14].

Application of microalgae as foliar sprays, seed primers, or soil amendments significantly enhances grain yields, root development, shoot dry matter, germination rates, carotenoid and chlorophyll accumulation, nitrate reduction, and overall plant height [15]. In addition, they produce allelopathic compounds that function as bioherbicides or biological control agents, positioning them as valuable tools for environmentally sustainable pest management [16].

Microalgae encompass both prokaryotic cyanobacteria (also known as blue-green algae) and eukaryotic green algae. These photosynthetic organisms play a vital role in converting atmospheric CO_2_ into organic biomass [9]. They are responsible for approximately 50% of Earth’s oxygen production by transforming sunlight into bioenergy and contributing to CO_2_ stabilization [17].

Additionally, microalgae play a vital role in mitigating climate change through carbon sequestration by absorbing atmospheric CO_2_ via photosynthesis [18]. In fact, for every kilogram of biomass produced, microalgae can sequester approximately 1.3 kg of CO_2_ [19]. Their carbon adsorption capacity is 10–50 times greater than that of terrestrial plants, without competing with food sources for humans or animals [20]. This efficiency is largely due to a specialized mechanism known as the carbon concentration mechanism (CCM). Through this process, the pyrenoid—an organelle located near the thylakoid membranes—elevates CO_2_ concentrations in its vicinity. This localized CO_2_ accumulation enhances the performance of ribulose-1,5-bisphosphate carboxylase/oxygenase (Rubisco), a key enzyme involved in photosynthesis that facilitates CO_2_ fixation. Despite its fundamental role, Rubisco exhibits a low affinity for CO_2_ due to its evolutionary adaptation to ancient high-CO_2_ and low-O_2_ environments. The pyrenoid helps to compensate this inefficiency by continuously creating a favorable microenvironment for enhanced carbon assimilation [21,22].

Given their multifunctional composition, microalgae are considered as a promising feedstock for both CO_2_ sequestration and bioenergy production [20]. However, scaling up algal cultivation for large-scale agriculture and wastewater treatment remains challenging. Species selection is crucial, as algal performance varies with the types of pollutants and environmental conditions [23]. Some microalgal species also show potential in phytoremediation, effectively removing contaminants such as heavy metals from polluted soils and contributing to land rehabilitation for agricultural purposes [24].

In this context, the circular bioeconomy paradigm has emerged, emphasizing sustainable production, harnessing solar energy, and converting renewable resources into valuable bioproducts [25]. Microalgae-based biotechnology plays a pivotal role in advancing this paradigm. These organisms can transform solid, liquid, and gaseous waste residues into nutrient-rich biomass for producing high-value bioproducts. The sequential extraction of such bioproducts using microalgae-based biorefinery offers a viable strategy to improve the economic feasibility of the process [26]. *Chlorella vulgaris* is able to eliminate pollutants from undiluted textile wastewater to below legal discharge limits, with 0.234–0.290 d^−1^ growth rates, 78–112.39 mg DW L^−1^ d^−1^ productivities, and valuable pigment production for circular economy applications [27]. The use of 20 g L^−1^ organic fertilizer combined with 20 mM urea for *Chlorella* sp. cultivation can enhance biomass productivity to 1.04 g L^−1^ d^−1^ and lutein content to 6.03 mg g^−1^ while reducing production costs by ~96–97%, and ≥1 μM lutein greatly reduced reactive oxygen species (ROS) in mammal cells subjected to blue-light irradiation [28]. In spite of their elevated production costs, *Chlorella* sp. and *Spirulina (Arthrospira)* sp., representing more than 90% of the world’s microalgal biomass production, are gaining wider utilization outside nutraceuticals in wastewater treatment, biofuel production, innovative medical treatments, and green industrial technologies [29].

This review highlights the diverse applications of microalgae in waste remediation, biofuel development, and biofertilizer production within the framework of a circular bioeconomy. It also explores current challenges, limitations, and future perspectives [30], underscoring the potential benefits of microalgal technologies in enhancing carbon sequestration, improving soil health, and advancing sustainable agriculture. Furthermore, it addresses the key barriers and future opportunities in adopting microalgae for climate-smart soil management practices.

## 2. Contribution to Soil Health Improvement

Microalgae are an effective tool in regenerative and sustainable agriculture due to their remarkable biological and biochemical capabilities for enhancing soil health [31]; microalgae serve diverse ecological functions, including biological nitrogen fixation, carbon sequestration, pollutant remediation, soil stabilization, and augmentation of organic matter [4].

Microalgal biomass contributes to soil enrichment through decomposition processes that increase organic matter content [9], improve soil structure [32], enhance water-holding capacity [33], and stimulate microbial enzymatic activity [31]. A key mechanism underlying these effects is the secretion of extracellular polymeric substances (EPSs), which bind soil particles, improve aggregate stability [32], reduce evaporation losses [33], and increase porosity [32].

Microalgae contribute significantly to soil aggregation not only through EPS secretion but also via physical entanglement, mucilage formation, and flagellar interactions. These mechanisms promote better gas exchange, water retention, erosion resistance, and overall soil fertility, all crucial for crop productivity [32]. While microaggregates (<250 μm) form through mineral interactions, microbial and algal exudates bind them into macroaggregates (>250 μm), making aggregation a key indicator of soil health [34]. Cyanobacteria and soil algae support topsoil stabilization by forming biocrusts through EPS secretion, trichome entanglement, and mucilaginous sheath formation, thereby enhancing soil texture and integrity [32,35].

Microalgae also secrete plant growth regulators such as gibberellins, cytokinins, and auxins to enhance root development and promote exudation [8,36]. These processes stimulate microbial proliferation and further contribute to aggregate formation [37]. Specific strains, including *Chlorococcum mexicana* and *C. sajao*, are known to improve soil aggregate stability in temperate agricultural environments [38]. Acidophilic species such as *Desmodesmus* and *Heterochlorella* form algal crusts on soil surfaces, stabilize acidic soils, and raise pH levels [39]. Chamizo (2018) demonstrated that both the nitrogen-fixing *Scytonema javanicum* and the non-N-fixer *Phormidium ambiguum* enhance EPS production and contribute to biocrust formation, improving soil integrity and erosion resistance [40].

EPS produced by cyanobacteria also bind soil particles and metal ions (Ca, Fe, and Zn), forming stable organo-mineral complexes that enhance aggregation [9,41]. *Chlamydomonas* species adhere to soil particles via electrostatic forces and flagella to facilitate aggregation [42]. Overall, cyanobacteria and green algae influence soil physical properties, promote root growth, and contribute to carbon sequestration through biochemical secretion and physical wrapping mechanisms [43,44].

Specific microalgae strains, in degraded or saline soils, improve the soil quality by enhancing organic matter, lowering pH, enhancing catalase, sucrase, and urease activities, boosting chlorophyll, and improving microbial performance [45]. In tomato cultivation, microalgae-based biofertilizers can substantially increase soil nutrients such as phosphorus by 27.4%, dissolved organic carbon by 231.3%, organic nitrogen by 403.4%, ammonium-N by 125.2%, nitrate-N by 215.6%, and magnesium by 73.4% [31,45,46,47,48,49,50,51].

Biofertilizers derived from microalgae are recognized as sustainable, affordable, and eco-friendly alternatives to synthetic inputs. Their versatility spans multiple ecosystems, from enhancing nitrogen fixation in tropical lowlands to mitigating erosion in temperate zones [4,7]. Widely used strains in paddy rice cultivation include *Nostoc*, *Anabaena*, *Tolypothrix*, and *Aulosira*, while symbiotic combinations such as *Azolla-Anabaena* and *Rhizobium* species are known to improve both soil fertility and crop performance [52,53]. In oil palm farming, microalgal biofertilizers have led to notable growth improvements [54]. Similarly, experiments with maize demonstrated that *Nostoc piscinale* increased humus content (a proxy for soil organic matter) by 17–20% compared to control conditions, indicating significant enhancement of soil fertility [55].

From this finding, it is clear that EPS-producing green algae, nitrogen-fixing cyanobacteria, and plant growth regulators are combined to form a multi-strain microalgal biofertilizer, releasing species to improve crop yields, salvage degraded soils, and store carbon in a variety of agricultural environments.

## 3. Contribution to Sustainable Agriculture

The application of microalgae such as *Chlorella vulgaris* and *Spirulina platensis* has shown notable improvements in soil quality and crop yields, with up to a 20.9% increase reported in rice cultivation [56]. In maize, microalgal treatments have resulted in enhanced germination rates, early-stage growth, and improved yield parameters [57]. For example, inoculation with *Monoraphidium* sp. in tomato (*Solanum lycopersicum*) resulted in a 32% increase in shoot biomass and a 12% rise in chlorophyll-a content, thereby improving photosynthetic efficiency [58]. Similarly, *Nostoc piscinale* positively influenced vegetative growth and grain yield in maize (*Zea mays*) [59]. In the case of *Anabaena* species, mass yield, nitrogen fixation, disease resistance, nutrient uptake, and soil fertility in wheat and other crops are significantly increased [60].

**Figure 1:**
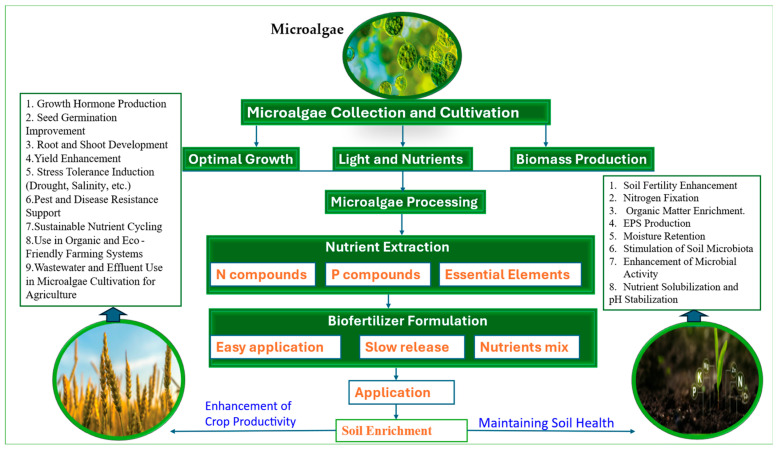
A schematic representation contribution of microalgae in soil improvement and sustainable agriculture.

A wheat field experiment showed that wastewater-grown microalgae plus compost increased microbial biomass carbon by 31.8–67.0%, cut nitrogen fertilizer needs by 25%, and improved grain nitrogen content (3.56%), plant dry weight (7.4–33.1%), spike weight (≤10%), and thousand-grain weight (5.6–8.4%) [61]. In Brazil, co-inoculation of *Chlorella vulgaris* with *Rhizobium tropici* and *Azospirillum brasilense* improved bean yields by 219.7 kg ha^−1^ in clay soil and 656.0 kg ha^−1^ in sandy soil, yielding 25.6% higher profits compared to nitrogen-fertilized controls [62] (Figure 1 and Table 1).

Cyanobacteria such as *Nostoc* and *Anabaena* have demonstrated the ability to reduce synthetic nitrogen fertilizer requirements by 25–50%, enhance microbial activity and crop yields by 5–25%, fix biologically 25–40 kg ha^−1^ of nitrogen, and minimize nitrogen leaching to around 7%, compared to 50% under conventional systems [63]. Their secretion of EPS significantly improves soil structure by 85–160% across loam, silty clay, and sandy soils, enhancing aeration, water infiltration, and moisture retention [64]. Moreover, co-inoculation with *Bacillus* sp. and other species increases microbial diversity and suppresses soil pathogens in continuous tomato cultivation, reinforcing the system’s resilience against biological stress [65,66].

Microalgae also act as potent bio-stimulants. Extracts from *Scenedesmus obliquus* have been reported to increase seed germination by 40%, auxin-like activity by 60%, and cytokinin-like activity by 187.5% in crops like mung bean and watercress [67]. Mixed algal treatments further improve shoot and root length, biomass accumulation, flower count, and overall yield, with increases of 46–57% relative to controls [68,69].

Microalgae can improve crop yields by 15.7–29.6%, reduce chemical fertilizer use, and restore degraded agroecosystems via nutrient cycling, nitrogen fixation, soil improvement, microorganism activation, and stress alleviation [70]. These findings confirm that microalgae are a robust, versatile, and environmentally sustainable resource for enhancing soil health, stress tolerance, and agricultural productivity. Figure 1 and Table 1 summarize how microalgae contribute to soil health improvement and sustainable agriculture.

**Table 1:** Contribution of microalgae in soil improvement and sustainable agriculture.

Species	Function/Method	Effect/Benefits	References
*Arthrospira platensis*, *Chlorella vulgaris*, *Nostoc muscorum*, *Anabaena azollae*, *Scenedesmus* spp., *Dunaliella salina*	Soil enrichment, EPS secretion, biomass decomposition	Enhance soil health, increase organic matter, improve soil structure, water-holding capacity, and microbial enzymatic activity	[4,9,31,32,33,37]
*Nostoc muscorum*, *Tolypothrix tenuis*, *Anabaena* spp.	EPS secretion (soil particle binding, mucilage, flagella)	Improve aggregation, porosity, reduce evaporation, enhance gas exchange, water retention, erosion resistance, and soil fertility	[32,33,34,44]
*Nostoc calcicole*, *Cyanobacteria*, *Scytonema* spp., *Anabaena* spp.	Biocrust formation (EPS, trichomes, mucilage)	Stabilize topsoil, improve soil texture, and integrity	[32,35,71]
*Nostoc commune*, *Tolypothrix distorta*, *Trichocoleus desertorum*, *Leptolyngbya frigida*, *Chlorella vulgaris*, *Nannochloropsis salina*, *Arthrospira platensis*, *Spirulina platensis*	Secretion of plant growth regulators (gibberellins, cytokinins, auxins) and seed germination	Enhance root development, stimulate microbial activity, and increase aggregate formation	[8,36,37,72]
*Chlorococcum mexicana*, *C. sajao*	EPS and soil aggregation	Improve soil aggregate stability in temperate agriculture	[38]
*Desmodesmus*, *Heterochlorella*	Acidophilic biocrust formation	Stabilize acidic soils, raise pH levels	[39]
*Scytonema javanicum* (N-fixer), *Phormidium ambiguum* (non-N-fixer)	EPS production, biocrust formation	Enhance soil integrity, erosion resistance	[40]
*Chlorella spp.*, *Scenedesmus* spp., *Nannochloropsis* spp., *Dunaliella salina*	Binding soil particles and metals (Ca, Fe, Zn)	Form organo-mineral complexes, enhance aggregation	[9,41]
*Chlamydomonas* spp.	Electrostatic/flagellar adhesion	Facilitate soil particle aggregation	[42]
*Tetradesmus obliquus and Chlorella sorokiniana*, *Chlorella pyrenoidosa*, *Azotobacter beijerinckii*, *Leptolyngbya* spp., *Dunaliella salina*	Soil improvement in degraded/saline soils	Enhance organic matter, lower pH, improve enzyme activity, and microbial performance	[45,73,74,75,76]
*Tribonema* spp., *Thermonaerobaculia*, *Subgroup_10*, *Sordariomycetes*, *Microascaceae*, *Pseudomonas*, *Togniniaceae*, *and Phaeoacremonium*, *Chlorella ellipsoidea*, *Arthrospira maxima*	Soil nutrient enrichment in tomato cultivation	↑ Phosphorus (27.4%), organic N (403.4%), ammonium-N (125.2%), nitrate-N (215.6%), Mg (73.4%)	[31,45,46,47,48,49,50,51]
*Nostoc*, *Anabaena*, *Tolypothrix*, *Aulosira*	Nitrogen fixation in paddy rice	Improve soil fertility, reduce synthetic inputs	[4,7,52,53]
*Azolla–Anabaena*, *Rhizobium* spp. (symbiotic)	Biofertilizer combinations	Enhance fertility, crop performance	[52,53]
*Nostoc piscinale*	Biofertilizer in maize	↑ Humus 17–20%, improve soil fertility and crop yield	[55,59]
*Chlorella vulgaris*, *Spirulina platensis*	Biofertilizer application	Improve soil quality, ↑ rice yield by 20.9%	[56]
*Monoraphidium* spp.	Tomato inoculation	↑ Shoot biomass 32%, ↑ Chlorophyll-a 12%	[58]
*Anabaena* spp.	Nitrogen fixation, growth regulator secretion	↑ Yield, nitrogen fixation, disease resistance, soil fertility (wheat and others)	[60]
Microalgae (*Chlorella* spp.) + compost (wheat field)	Wastewater-grown algal application	↑ Microbial biomass C (31.8–67%), ↓ fertilizer use 25%, ↑ grain N (3.56%), ↑ yield components	[61,77]
*Chlorella vulgaris* + *Rhizobium tropici* + *Azospirillum brasilense*	Co-inoculation (bean cultivation)	↑ Yield (219.7–656 kg ha^−1^), ↑ profit by 25.6%	[62]
*Nostoc* spp., *Anabaena*	EPS secretion, N fixation	↓ Fertilizer use by 25–50%, ↑ microbial activity 5–25%, fix 25–40 kg N ha^−1^, reduce leaching	[63,64]
*Burkholderia vietnamiensis* + *Trichoderma harzianum*	Co-inoculation in tomato	↑ Microbial diversity, suppress pathogens, enhance resilience	[65,66]
*Scenedesmus obliquus*	Bio-stimulant activity	↑ Germination 40%, auxin-like activity 60%, cytokinin-like activity 187.5%	[67]
*Trichormus variabilis*, *Auxenochlorella pyrenoidosa*, *Spirulina platensis* *Anabaena* spp., *Tribonema* spp., *Chlorella vulgaris*	Biofertilizer and soil restoration	↑ Crop yields 15.7–29.6%, ↓ fertilizer use, restore degraded soils	[70]

Note: (↑) Increase and (↓) Decrease.

## 4. Contribution to Carbon Sequestration and Climate Change Mitigation

Microalgae play a pivotal role in the global carbon cycle and carbon sequestration, contributing both to natural ecosystems and engineered systems. Through biological carbon fixation, they significantly influence atmospheric CO_2_ concentrations [78]. Marine microalgae, especially phytoplankton such as diatoms, are responsible for approximately 50 gigatons (Gt) of CO_2_ fixation annually, representing half of Earth’s total biological carbon assimilation [79]. Remarkably, diatoms alone account for 20% of global CO_2_ sequestration, a capacity equivalent to that of all the world’s rainforests combined [80].

Their ability to capture atmospheric CO_2_ is further amplified by their tolerance to other greenhouse gases, including hydrocarbons (HC), sulfur dioxide (SO_2_), methane, and nitrogen oxides (NO_x_). It is estimated that microalgae can absorb up to 77% of total greenhouse gas emissions [20], operating with a carbon capture efficiency 10–50 times higher than terrestrial plants [81].

Photosynthesis is their primary mechanism for capturing carbon. Benefiting from a high surface area-to-volume ratio and accelerated growth rates, microalgae can fix CO_2_ up to 50 times faster than land flora [82,83]. Certain species, such as *Chlorella vulgaris*, have demonstrated sequestration rates of 1.6–2 tons of CO_2_ per ton of biomass produced [84]. In photobioreactor and open raceway pond systems, biomass yields can reach up to 82 tons per hectare annually [85]. Emerging technologies, including spray absorption towers, have enhanced CO_2_ fixation performance, achieving improvements of 50%, compared to the 11.17% efficiency of conventional bubbling systems [86].

Research in China’s karst wetlands indicates that microalgae can fix over 4200 tons of carbon annually, with 28.7% of bicarbonate converted into organic carbon via photosynthesis [87], underscoring their effectiveness as natural carbon sinks in aquatic and wetland environments. Global estimates of microalgal biomass production indicate 93,756 tons in 2010, 87,000 tons in 2018, and 56,465 tons in 2019 [88], which correlates with CO_2_ sequestration totals of approximately 187,500 tons, 174,000 tons, and 112,900 tons, respectively [20,89].

Engineered systems designed for industrial-scale CO_2_ capture include photobioreactors, bioenergy facilities, and biochar platforms [90], which can be integrated with factories and power plants to extract CO_2_ directly from flue gas emissions [91]. The harvested biomass supports the production of biofuels [19], bioplastics [92], fertilizers [90], and biochar [90], a soil amendment that not only enhances long-term carbon storage but also reinforces the circular carbon economy [91].

Under optimal conditions in photobioreactors, *Chlorella* species can achieve CO_2_ uptake rates of 160–175 mg of biomass per liter per day [93]. Some systems have reached capture efficiencies of 93.7% [94], with comparable results obtained in continuously stirred tank reactors, which report up to 178 mg L^−1^ d^−1^ and 96% CO_2_ removal efficiency [95].

**Figure 2:**
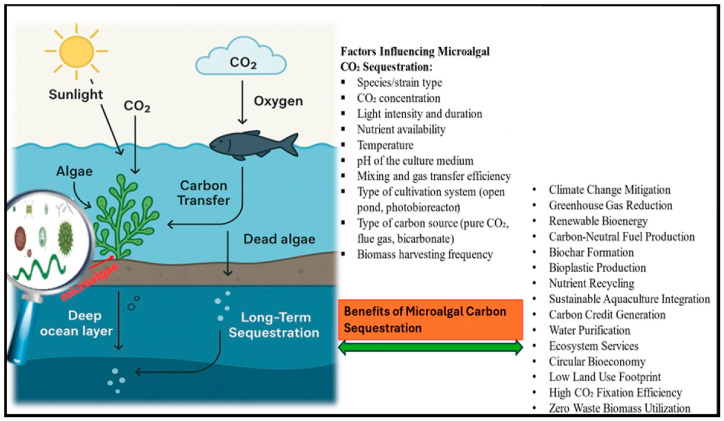
A schematic representation of the contribution of microalgae to carbon sequestration.

In agricultural contexts, microalgae provide synergistic benefits, enhancing crop productivity while contributing to carbon sequestration and mitigating greenhouse gas emissions. One study in hawthorn orchards found that microalgal biofertilizers increased fruit yields by 29.6%, improved soil organic carbon content, and maintained stable greenhouse gas emission levels [70]. Figure 2 and Table 2 summarize how microalgae contribute to carbon sequestration and climate change mitigation.

## 5. Contribution to Environmental Benefits

As previously noted, atmospheric CO_2_ can be effectively sequestered by microalgae, which convert it into biomass through photosynthesis [9]. This biological process is crucial for reducing greenhouse gas emissions and mitigating climate change. Due to their adaptability to aquatic environments and rapid growth rates, microalgae fix atmospheric CO_2_ more efficiently than terrestrial plants [18]. They are particularly effective at absorbing excess CO_2_ from industrial emissions while simultaneously producing oxygen [98]. For example, in photobioreactors, microalgae utilize CO_2_ from flue gases and other waste streams to generate valuable biomass [86].

Algal biomass serves as a sustainable alternative to fossil fuels and synthetic fertilizers, helping to reduce emissions and dependency on resources [99,100]. Biofuels derived from microalgae enable closed-loop carbon recycling by reusing absorbed CO_2_ and generating oxygen, offering a renewable energy source with significantly lower carbon emissions compared to conventional fossil fuels [83].

Additionally, microalgae can absorb airborne pollutants such as nitrogen oxides (NO_x_) and sulfur oxides (SO_x_), contributing to reductions in smog and acid rain. These pollutants are metabolized in biofiltration systems, making microalgae effective tools for air purification in urban and industrial settings [101].

Microalgae cultivation has shown promise in areas affected by soil degradation or desertification, improving agricultural productivity and restoring soil fertility [102]. Species adapted to saline environments have been successfully grown in brackish water and degraded soils to enhance organic matter content and improve soil structure [103].

Thanks to their resilience, microalgae thrive in diverse environmental conditions, including arid, nutrient-poor, and saline soils [103]. By assimilating excess nutrients such as phosphorus and nitrogen, which contribute to eutrophication, microalgae efficiently purify wastewater [104]. They also enhance water quality through the biosorption and bioaccumulation of heavy metals, such as cadmium, lead, and mercury [24,105]. These features make microalgae especially suitable for agricultural development in regions facing water scarcity or high salinity [106].

Furthermore, microalgae can be processed into biodegradable and renewable products such as building materials, textiles, and bioplastics, advancing circular economy principles and reducing long-term pollution [107].

Microalgae-powered systems can capture industrial CO_2_, purify air and wastewater, restore degraded soils, and produce renewable fuels, fertilizers, and biodegradable materials, creating a single, self-sustaining solution for climate, energy, and resource challenges.

**Figure 3:**
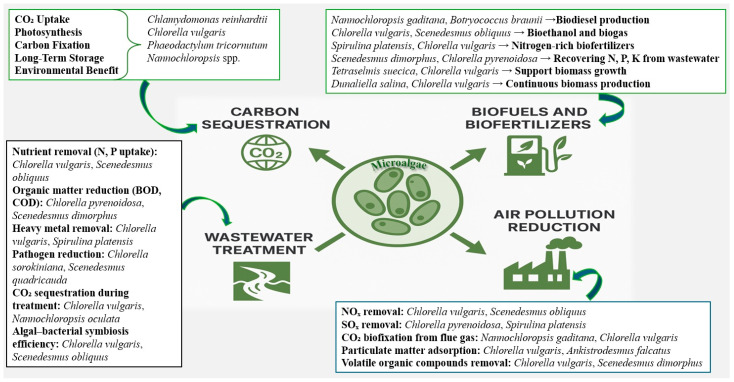
Roles of microalgae in climate mitigation and ecosystem restoration. Microalgae sequester atmospheric CO_2_, purify wastewater, improve soil health in degraded environments, and produce renewable bioproducts supporting sustainable agriculture and circular bioeconomy strategies.

Figure 3 and Table 3 summarize how microalgae sequester atmospheric CO_2_, purify wastewater, improve soil health in degraded environments, and produce renewable bioproducts supporting sustainable agriculture and circular bioeconomy strategies.

## 6. Contribution to Circular Economy and Waste Valorization

Microalgae such as *Chlorella* and *Scenedesmus* are highly efficient in wastewater treatment, capable of removing nutrients like nitrate, phosphate, and ammonium [108], as well as heavy metals (Cu, Zn, Pb, Cr, Cd), dyes, and organic pollutants, with removal rates ranging from 90% to 98% [109]. By assimilating these compounds into their biomass, they purify water while generating a valuable feedstock [26]. Additionally, microalgae utilize CO_2_ from flue gases to accelerate growth, transforming industrial emissions into useful bioresources [110]. For example, *Micractinium pusillum* has demonstrated CO_2_ fixation rates of ~137 mg L^−1^day^−1^, lipid yields of nearly 32%, and biomass production of 1.3 g L^−1^, even when cultivated directly in flue gas streams [111].

This capacity supports co-treatment strategies, turning pollutants and effluents into productive outputs [112]. Integrated biorefineries offer a sustainable solution, producing biofuels such as biodiesel, biogas, and bioethanol, alongside high-value co-products including omega-3 oils, proteins, phycobiliproteins, biofertilizers, and bioplastics, aligning with a zero-waste valorization model [26]. Certain strains like *Schizochytrium* sp. and *Nannochloropsis* spp. exhibit lipid content exceeding 45% dry weight. When cultivated on food waste hydrolysates, they can reach 49% lipid accumulation and 32% FAME yield [112].

The cultivation of *Nostoc* sp., *Arthrospira platensis*, and *Porphyridium purpureum* in industrial wastewater has demonstrated removal rates of 98% COD, 94% nitrogen, and 100% phosphate, with phycocyanin yields up to 103 mg g^−1^ dry weight [113].

Residual biomass can be converted into biochar, which improves soil fertility, enhances water retention, and contributes to long-term carbon sequestration—key components of circular and net-zero strategies [114]. In the EU Horizon project WWTBP-by-Microalgae, *Arthrospira platensis* treated brewery effluents by removing 90% of nutrients and CO_2_, producing phycocyanin pigments valued at EUR 221 kg^−1^, biogas (93 mL CH_4_ g^−1^ VS), and biochar [115].

Pilot-scale systems demonstrate nitrogen and phosphorus removal above 90% in both municipal and industrial wastewater treatments [116]. In warmer regions, hybrid pond systems for biogas and biofertilizer production offer more sustainable alternatives to conventional wastewater methods [105]. Cascading biorefinery models maximize resource recovery beginning with lipid extraction, followed by protein, pigment, and carbohydrate recovery [112,117].

Economic viability remains a challenge but is improving. In Almeria, Spain, a tubular photobioreactor using *Scenedesmus almeriensis* reported a cost of ~EUR 69 kg^−1^ dry biomass, projected to fall to EUR 12–13 kg^−1^ at 200 t y^−1^ scale [118]. In Italy, a 1-hectare Green Wall Panel system with *Tetraselmis suecica* reported costs of EUR 12.4 kg^−1^, reducing to EUR 5.1 kg^−1^ at 100 ha scale and potentially EUR 3.2 kg^−1^ under optimal climate conditions [119]. Scale-up involves logistical complexities in harvesting, drying, and extraction, requiring optimization to lower energy demand. Additionally, regulatory gaps and limited market infrastructure hinder widespread adoption [120].

Nonetheless, industry outlooks remain positive. According to Fortune Business Insights (June 2025), the global microalgae market is expected to grow at a CAGR of 7.29%, rising from USD 782.6 million in 2024 to USD 1.38 billion by 2032 [121]. Table 4 summarizes how microalgae integrate CO_2_ mitigation, wastewater purification, high-value product generation, and validation of the circular economy, alongside current limitations in cost, scaling, and regulation.

## 7. Challenges, Limitations, Solutions, and Future Directions

The widespread application of microalgae faces considerable technical, economic, and environmental hurdles that hinder its commercial deployment across various sectors, including biofuels, food, pharmaceuticals, and wastewater treatment [124,125]. While low-cost open raceway ponds are easy to implement, they are prone to contamination, have low productivity, and offer limited environmental control, resulting in inconsistent yields [23,126,127]. In contrast, photobioreactors (PBRs) ensure operational control and high biomass productivity, yet their elevated capital and energy costs restrict scalability [128,129,130]. Additional challenges in closed systems include insufficient light penetration in dense cultures and photoinhibition, which reduces efficiency [91,131,132].

Downstream processing remains complex and energy-intensive, as extracting pigments, lipids, and proteins often requires cell wall disruption that can affect microalgal viability [133,134]. Microalgae also exhibit high sensitivity to variations in temperature, light, salinity, and pH, confining large-scale outdoor cultivation to specific geographic zones [135,136]. From an economic standpoint, the cost per liter of algal biofuels and derivatives still exceeds that of other biomass sources and fossil alternatives—even when factoring in co-products [137,138].

Despite these constraints, microalgae show strong promise as biofertilizers and soil amendments in regenerative agriculture [4,7,139]. Species such as *Spirulina* and *Chlorella* are rich in nitrogen, phosphorus, potassium, amino acids, and micronutrients. These compounds improve soil structure, promote microbial communities, and boost nutrient availability [4,140,141]. Their application reduces reliance on synthetic fertilizers, preventing contamination of soil and water systems [141,142,143]. Integrating algal biomass into biochar further enhances carbon retention, presenting a natural method for long-term soil carbon storage [19,144].

Large-scale microalgae cultivation in PBRs or open ponds can be coupled with industrial CO_2_ capture systems, thereby aiding in emission reductions from fossil fuel combustion and manufacturing processes [145,146]. In sustainable farming, microalgae also contribute to stress resilience and pest control through the production of natural antimicrobial and antifungal metabolites [8,147]. Their ability to grow on non-arable land or wastewater positions them as resource-efficient inputs that do not compete with food crops [148]. This circular approach reinforces their potential in climate-smart agriculture, placing microalgae at the core of resilient agroecosystems [149,150].

The advancement of genetic engineering and synthetic biology offers pathways to develop stress-tolerant strains with enhanced metabolite yields and growth rates [151,152,153]. Parallel improvements in cultivation and harvesting techniques, focusing on energy savings and cost reduction, are vital for scaling operations. Additionally, integrated algal biorefineries should be developed to produce biofertilizers, biofuels, feedstocks, bioplastics, and nutraceuticals, ensuring economic feasibility [154,155,156,157].

Supportive policies will be instrumental in accelerating adoption: Financial incentives, increased research funding, and regulatory frameworks are needed to safeguard ecological integrity [158]. Public engagement and agricultural education will also be key in fostering acceptance at the community level. Through coordinated scientific and societal efforts, microalgae can become a cornerstone in climate-resilient agriculture, enabling carbon neutrality, soil restoration, and a global transition to sustainable bioeconomy systems (Table 5).

From the discussion, it is clear that *Spirulina* spp. and *Chlorella* spp. are nutrient-rich for biofertilizers, stress-tolerant strains like engineered *Chlorella vulgaris* and *Dunaliella salina* suit harsh conditions, and high-biomass species *Nannochloropsis. Scenedesmus* enables biofuels and CO_2_ capture, *Haematococcus* and *Spirulina* provide bioactive compounds, versatile *Chlorella* and *Nannochloropsis* support multi-product biorefineries for a circular bioeconomy [4,26].

## 8. Conclusions

Microalgae are increasingly recognized as a versatile and sustainable biological resource, offering transformative potential across agriculture, environmental remediation, energy production, and industrial biotechnology. Their exceptional ability to sequester atmospheric CO_2_, eliminate pollutants, purify wastewater, and rehabilitate degraded soils positions them as a strategic asset in confronting the interlinked global crises of climate change, soil degradation, and food insecurity. In agriculture systems, microalgae act as biofertilizers and biostimulants, contributing to enhanced crop productivity, improved soil health, and more efficient nutrient cycling. These benefits are achieved while simultaneously reducing dependence on synthetic agrochemicals, thereby promoting more ecologically sound farming practices. Moreover, their capacity to thrive on non-arable land and utilize waste and carbon streams underscores their suitability for integration into circular bioeconomy frameworks. The emergence of algal biorefineries presents a scalable and economically viable approach to the production of high-value commodities, including biofuels, bioplastics, pharmaceuticals, and functional foods. These systems support zero-waste valorization strategies and offer promising avenues for diversifying revenue streams within sustainable industries. However, despite these advantages, widespread commercialization remains constrained by several challenges—namely, energy-intensive downstream processing, cultivation limitations, elevated production costs, and insufficient regulatory support. Encouragingly, recent innovations in genetic engineering, hybrid cultivation platforms, and cascading extraction technologies are paving the way to overcome these barriers. Realizing the full potential of microalgae will require targeted investments in research and development, infrastructure, and the establishment of coherent policy frameworks. Equally important are public awareness, interdisciplinary collaboration, and educational initiatives to foster acceptance and long-term integration. With coordinated global efforts, microalgae stand poised not only to revolutionize agricultural paradigms but also to play a pivotal role in advancing carbon neutrality, restoring ecological balance, and building a resilient, bio-based economy for future generations.

## Figures and Tables

**Table 2:** Contribution of microalgae to carbon sequestration and climate change mitigation.

Species/System	Function/Method	Effect/Benefits	References
*Phaeodactylum tricornutum*, *Thalassiosira pseudonana*, *Scenedesmuss obliquss*, *Duniella tertiolecta*, *Chlorella vulgaris*, *Phormidium* spp., *Amicroscopica negeli*, *Chlorococcum littorale*, *Isochrysis* spp., *Botryococcus braunii*, *Haematococcus pluvialis*	Biological carbon fixation (photosynthesis)	Fix ~50 Gt CO_2_ annually, diatoms alone account for ~20% of global CO_2_ sequestration and highest CO_2_ fixation rate 0.35 g CO_2_ L d^−1^ after acclimatization	[78,79,80,96,97]
*Botryococcus braunii*, *Dunaliella salina*, *Scenedesmus obliquus*, *Arthrospira platensis*	Absorption of greenhouse gases (CO_2_, HC, SO_2_, CH_4_, NO_x_)	Capture up to 77% of total GHG emissions; carbon capture efficiency 10–50× higher than terrestrial plants	[20,81]
*Chlorella vulgaris*, *Scenedesmus obliquus*, *Dunaliella salina*, *Haematococcus pluvialis*, *Coelastrella saipanensis*	Photosynthesis (high surface area-to-volume ratio, fast growth)	Fix CO_2_ up to 50× faster than land plants	[82,83]
*Chlorella vulgaris*	Biomass-based carbon sequestration	1.6–2 tons CO_2_ per ton biomass; yields up to 82 tons biomass ha^−1^ y^−1^ in open ponds or photobioreactors	[84,85]
Photobioreactors (spray absorption towers)	Enhanced CO_2_ capture efficiency	Up to 50% improvement vs. 11.17% in bubbling systems	[86]
Karst wetland microalgae (China)	Natural aquatic carbon sink (photosynthesis)	Fix ~4200 tons C annually; convert 28.7% of bicarbonate into organic carbon	[87]
Global microalgal biomass (*Chlorella* spp.), *Scenedesmus obliquus*, *Nannochloropsis gaditana*, *Botryococcus braunii*, *Spirulina platensis*	Biomass production linked to CO_2_ sequestration	93,756 t (2010), 87,000 t (2018), 56,465 t (2019) → ~187,500 t, 174,000 t, 112,900 t CO_2_ sequestered respectively	[20,88,89]
Engineered systems (photobioreactors, bioenergy, biochar platforms)	Industrial-scale CO_2_ captured from flue gases	Capture efficiencies up to 93.7%; biomass used for biofuels, bioplastics, fertilizers, biochar (long-term carbon storage)	[19,90,91,92,93,94,95]
*Chlorella* spp. (under optimal reactor conditions), *Scenedesmus obliquus*, *Nannochloropsis gaditana*, *Spirulina platensis*, *Botryococcus braunii*	Biomass productivity	160–175 mg L^−1^day^−1^ biomass yield; up to 96% CO_2_ removal efficiency	[93,94,95]
Agricultural applications (hawthorn orchards with microalgal biofertilizers), *Chlorella* spp., *Scenedesmus* spp., *Spirulina platensis*	Soil enrichment and crop support	+29.6% fruit yield; improved soil organic C; stable GHG emission levels	[70]

**Table 3:** Contribution of microalgae to environmental benefits.

Environmental Benefit	Effect/Application	References
Atmospheric CO_2_ sequestration	Conversion of CO_2_ into biomass via photosynthesis; reduces greenhouse gases and mitigates climate change	[9,18,98]
Industrial CO_2_ capture	Absorbs CO_2_ from flue gases and waste streams in photobioreactors while producing oxygen	[86]
Renewable bioenergy	Biomass used for biofuels → enables closed-loop carbon recycling, lower emissions vs. fossil fuels	[83,99,100]
Air purification	Absorption and metabolism of NO_x_ and SO_x_; reduces smog and acid rain	[101]
Soil restoration	Improves fertility and productivity in degraded soils; enhances organic matter content and structure in saline/brackish soils	[102,103]
Adaptability to harsh conditions	Thrives in arid, nutrient-poor, and saline environments; suitable for sustainable agriculture in water-scarce regions	[103,106]
Wastewater treatment	Assimilates excess nitrogen and phosphorus (reduces eutrophication); enhances water quality	[104]
Heavy metal removal	Biosorption and bioaccumulation of Cd, Pb, Hg, and other metals from polluted water	[24,105]
Biodegradable products	Processed into bioplastics, textiles, and building materials; reduces plastic pollution and supports circular economy	[107]

**Table 4:** Contribution of microalgae to circular economy and waste valorization.

Species/System	Function/Method	Effect/Benefits	References
*Chlorella*, *Scenedesmus*	Wastewater treatment	Remove nitrate, phosphate, ammonium, heavy metals (Cu, Zn, Pb, Cr, Cd), dyes, organic pollutants (90–98% removal); generate valuable biomass feedstock	[26,108,109]
*Micractinium pusillum*	Cultivation in flue gases	CO_2_ fixation ~137 mg L^−1^ d^−1^; lipid yield ~32%; biomass 1.3 g L^−1^	[110,111]
*Multi-strain biorefineries:* **Freshwater** *Chlorella vulgaris, Scenedesmus obliquus, Ankistrodesmus falcatus, Desmodesmus* spp. **Saltwater** *Tetraselmis suecica,* *Nannochloropsis gaditana* *Dunaliella salina, Isochrysis galbana*	Co-treatment of pollutants and effluents	Production of biodiesel, biogas, bioethanol, omega-3 oils, proteins, pigments, biofertilizers, bioplastics (zero-waste model)	[26,112]
*Schizochytrium* sp., *Nannochloropsis* spp.	Cultivation on food waste hydrolysates	>45% lipid content; up to 49% lipid accumulation; 32% FAME yield	[112]
*Nostoc* sp., *Arthrospira platensis*, *Porphyridium purpureum*	Industrial wastewater treatment	COD removal 98%, nitrogen 94%, phosphate 100%; phycocyanin yields up to 103 mg g^−1^	[113]
Residual biomass (*Chlorella vulgaris*)	Biochar production	Biomass productivity of 0.87 g L^−1^ day^−1^, improves soil fertility, water retention, and long-term carbon sequestration	[114,122,123]
*Arthrospira platensis* (EU Horizon project)	Brewery effluent treatment	Removed 90% nutrients and CO_2_; produced phycocyanin (EUR 221 kg^−1^), biogas (93 mL CH_4_ g^−1^ VS), biochar	[115]
Pilot-scale systems (municipal and industrial wastewater)	Hybrid ponds/bioreactors	>90% N and P removal; production of biogas and biofertilizers	[105]
Cascading biorefinery models	Sequential biomass valorization	Lipid → protein → pigment → carbohydrate recovery	[112,117]
*Scenedesmus almeriensis* (Spain)	Tubular photobioreactor	Biomass cost ~EUR 69 kg^−1^, projected EUR 12–13 kg^−1^ at 200 t y^−1^ scale	[118]
*Tetraselmis suecica* (Italy, Green Wall Panel system)	Large-scale cultivation	Biomass cost EUR 12.4 kg^−1^ (1 ha), reduced to EUR 5.1 kg^−1^ (100 ha), potentially EUR 3.2 kg^−1^ optimal	[119]
Global microalgae industry	Market outlook	Expected growth from USD 782.6 M (2024) to USD 1.38 B (2032), CAGR 7.29%	[121]
Challenges	Scale-up, regulation, logistics	High energy demand in harvesting/drying/extraction; regulatory and infrastructure gaps hinder adoption	[120]

**Table 5:** A comprehensive table compiles all algal species mentioned throughout the paper, alongside their documented uses and purposes.

Species	Benefits	References
*Chlorella vulgaris*	Removes pollutants from textile wastewater below legal limits; growth rate 0.234–0.290 d^−1^; productivity 78–112.39 mg DW L^−1^ d^−1^; pigment production; carbon sequestration 1.6–2 t CO_2_ per t biomass; improves rice yield (20.9%); co-inoculation enhances bean yield/profit	[27,56,62,84]
*Chlorella* sp.	Cultivation with organic fertilizer + urea increases biomass (1.04 g L^−1^ d^−1^) and lutein (6.03 mg g^−1^); reduces costs by 96–97%; lutein reduces ROS in mammal cells	[28]
*Spirulina (Arthrospira)* sp.	Major contributor to global biomass (>90%); used in nutraceuticals, wastewater treatment, biofuel, medicine, and green technologies; improves soil and crop yield	[29,56,115]
*Chlorococcum mexicana, C. sajao*	Improve soil aggregate stability in temperate agriculture	[38]
*Desmodesmus, Heterochlorella*	Acidophilic; stabilize acidic soils and raise pH	[39]
*Scytonema javanicum*	Nitrogen-fixer; enhances EPS production, biocrust formation, and soil stability	[40]
*Phormidium ambiguum*	Non-N-fixer; contributes to EPS production and biocrusts for erosion resistance	[40]
*Chlamydomonas* spp.	Soil particle aggregation via electrostatic forces and flagella	[42]
*Nostoc* spp.	Nitrogen fixation in paddy rice; reduces synthetic N fertilizer by 25–50%; improves soil fertility; enhances maize fertility (humus ↑ 17–20%); EPS improves soil structure	[4,52,53,55,59,63,64]
*Anabaena* spp.	Nitrogen fixation; improves crop yield, disease resistance, and soil fertility (wheat, maize, rice); symbiosis with Azolla enhances soil fertility	[52,53,60,63]
*Tolypothrix* spp.	Nitrogen fixation in paddy rice cultivation	[52,53]
*Aulosira* spp.	Nitrogen fixation in paddy rice	[52,53]
*Azolla–Anabaena symbiosis*	Biofertilizer combination improves soil fertility and crop growth	[52,53]
*Monoraphidium* sp.	Tomato inoculation → shoot biomass ↑ 32%, chlorophyll-a ↑ 12%	[58]
*Scenedesmus obliquus*	Extracts act as bio-stimulants: ↑ germination (40%), auxin-like (60%), cytokinin-like (187.5%); also, wastewater nutrient removal	[67,108,109,115]
*Micractinium pusillum*	Grows on flue gases; CO_2_ fixation ~137 mg L^−1^ d^−1^; lipid yield ~32%; biomass 1.3 g L^−1^	[111]
*Schizochytrium* sp.	High lipid content (>45%); up to 49% lipid accumulation; biodiesel and omega-3 production	[112]
*Nannochloropsis* spp.	High lipid producers (>45% DW); biofuels and multiproduct biorefinery	[112]
*Porphyridium purpureum*	Industrial wastewater treatment; phycocyanin yield 103 mg g^−1^ DW	[113]
*Tetraselmis suecica*	Large-scale cultivation; biomass cost reduction to EUR 3.2 kg^−1^ under optimal conditions	[119]
*Haematococcus* sp.	Bioactive compounds (e.g., astaxanthin)	[26]
*Arthrospira platensis, Chlorella vulgaris, Nostoc muscorum, Anabaena azollae, Scenedesmus* spp., *Dunaliella salina, Nostoc calcicole, Scytonema* sp.	Soil enrichment, EPS secretion, carbon sequestration, wastewater purification, pollutant remediation, biocrust formation, biofertilizers, and bioplastics	[4,9,26,31,32,33,70,104,107]

Note: (↑) Increase.

## Abbreviations

EPS	Extracellular polymeric substances
CO_2_	Carbon dioxide
CCM	Carbon concentration mechanism
Gt	Gigatons
HC	Hydrocarbons
SO_2_	Sulfur dioxide
PHB	Polyhydroxybutyrate
PLA	Polylactic acid
PBR	Photobioreactor
ROS	Reactive oxygen species
